# A critical review of heat pump adoption in empirical and modeling literature

**DOI:** 10.1016/j.isci.2024.111666

**Published:** 2024-12-19

**Authors:** Narasimha D. Rao, Mohammad R.K. Siam, Tami C. Bond

**Affiliations:** 1 Yale School of the Environment, New Haven, CT 06883, USA; 2 International Institute for Applied Systems Analysis, Laxenburg 2361, Austria; 3 Colorado State University, Fort Collins, CO 80523, USA

**Keywords:** Energy policy, Engineering, Energy systems

## Abstract

Household electrification is an important pillar of decarbonization in the US and requires the rapid adoption of electric heat pumps. Household energy models that project adoption rates do not represent these decisions well. To what extent are they limited by fundamental knowledge gaps, or is there scope to incorporate insights from the social science literature? We review the energy modeling and social science literature on heating equipment adoption to synthesize our understanding of adoption decisions, to identify best practices on representing decision-making behavior among energy models, and to suggest model improvements. At the most aggregated level, market allocation models divide market shares among different technologies by considering a single representative household, ignoring heterogeneity among the actors. Energy-system models and agent-based models can include some disaggregation. Adoption decisions include two stages, one to retire existing equipment, and to select the preferred technology. Equipment breaking down, price shocks, and moving to a new house promote entering the first stage, but these factors are not widely explored in surveys. The empirical literature reveals considerable heterogeneity in what matters to people in choosing technology. Even cost considerations, which are the most widespread, vary in the components and the manner in which they enter decisions. Other considerations include comfort and reliability; whether decision-makers are urban, young and educated; and how adopters perceive novel technologies. However, the relative strengths of these factors and how they vary across the US population are not known. Modelers can make incremental structural improvements such as separating the two decision stages, differentiating household groups, and incorporating changing household perceptions with market maturation. However, they cannot ground these in reality without considerable new fieldwork on decision-making processes and their variation across the population.

## Teaser

Classification Description: Energy policy; Engineering; Energy systems

## Introduction

How realistic are energy model projections in reflecting households’ decisions around changing heating equipment? To what extent are they limited by fundamental knowledge gaps, or is there scope to incorporate insights from the social science literature? These questions are the subject of this critical review. Household electrification is widely seen as the means of decarbonizing household fossil fuel use for cooking and heating.^1^ In 2021 the share of home heating in energy demand and greenhouse gas (GHG) emissions in the United States and Europe was ∼8% and ∼11%, respectively. Household electrification entails extensive retrofits to the existing building stock, primarily to replace fossil fuel-based heating with electric heat pumps (EHPs). The US Government’s Long-term Strategy for decarbonization requires that EHP would have to comprise 60 percent of heating equipment sales by 2030^2^. For the timely achievement of technology penetration targets, the replacement of existing equipment is critical. Gas furnaces and boilers may be used for up to 25 years on average.^3^ A rapid transition requires shortening this horizon. This would require that upon retirement households make the leap to embrace new, potentially unfamiliar technologies. EHP sales are gathering momentum—in the US, for the first time, EHP sales overtook those of gas furnaces in 2022.^4^ However, adoption rates in the Southeast and West dominate sales.^5^ While the Inflation Reduction Act (IRA) does allocate funds for home improvement, the EHP rebates may not be sufficient to tip enough households to achieve national electrification goals,^6^ and the incentives for efficient gas heaters undermine EHP incentives. Understanding the effectiveness of policies to shorten retirement horizons and motivate selection requires that we know how households make decisions. Likewise, if household choices inherently limit rates of future change and thus set emissions, this information needs to return to models of future climate mitigation.

Energy models typically forecast energy demand, energy supply and GHG emissions for the US and/or the world under different scenarios of socioeconomic futures, policy, and technology evolution. Household energy models would be useful to policymakers for guiding policy design if they reflected how households make decisions. Models could test the efficacy of existing incentives, evaluate new ones and examine trade-offs among different policy objectives and social impacts of phasing out fossil fuel heating appliances. For instance, one might want to know equity and efficiency trade-offs of EHP rebates to low-income communities. However, households are typically represented in energy models as single economic representative economic agents, or in a few cases as income-differentiated groups.^7^ Models typically allocate market shares to different competing technologies based on economic competitiveness, ignoring household heterogeneity and contextual and other behavioral factors. The question for future model development is whether there is sufficient and generalizable knowledge in the literature to justify developing, quickly, the next generation of national energy model scenarios to inform policy making.

This study explores the state of knowledge in the social science literature on modeling households’ decision-making around EHP adoption and makes recommendations for future directions in modeling. We focus on lessons for the US market but draw on literature in both the US and Europe, because of the longer history and popularity of EHP use in the latter case. We focus specifically on adoption decisions for EHPs (*extrinsic* decisions, in the economics parlance), rather than operational choices (*intrinsic* decisions), such as thermostat setpoints.

Notably, several recent review articles have focused on the general topic of identifying gaps between the social sciences and modeling. Most of these indeed motivate and provide a starting point for this article. Chadwick et al.^8^ review the state of knowledge in the social sciences on adoption decisions for home improvement in general, but do not address modeling. Haiskanen and Matchoss^9^ review adoption criteria for a number of residential renewable technologies, which we refer to later. Gaur et al. in their review of EHP technologies provide some insights on adoption barriers, also discussed later.^10^ Trutneveyte et al.^1^ in their review of how social science is represented in models suggest that most energy models tend to bridge or iterate between social science insights and models using exogenous assumptions and scenarios. They suggest that future modeling efforts should merge them through structural modifications to behavioral models based on generalizable empirical research. However, they address household technology adoption only to the extent of electric vehicles. Mastrucci et al.^11^ review the state of the art in modeling demand-driven energy transformations, including household behavior in models, and reach similar conclusions. They attribute the simplicity in modeling household behavior to the lack of granularity in representing households.

Krumm et al.^12^ come closest to this article in reviewing behavioral decisions around socio-technical transitions in European energy models. They provide similar recommendations to Trutneveyte et al. and Mastrucci et al. for future modeling, and in addition provide useful insights on the required research process that would enable deeper integration. They also find agent-based models (ABMs) to be the most promising direction to achieve this purpose. However, they do not study US energy models, nor do they delve deeply into the substance of household adoption behavior. In this article we explore the feasibility of the recommendations from these articles for EHP adoption, including structural changes to energy models to endogenize behavioral drivers and better representing household heterogeneity. We systematically review the empirical literature on EHP adoption and best practices in energy model projections to address these questions.

In summary, none of the previous reviews address the specific shortcomings in how decisions around retrofitting home heating equipment with EHPs are modeled and the knowledge that can be harnessed from the social sciences for that purpose. As noted in Strazzera et al.’s recent review, “a notable research gap emerging from this review is the need for a more nuanced understanding of how individual characteristics and contextual factors interact to influence the adoption of energy-efficient heating and cooling systems.”^13^ Our main contribution is to show that what may be viewed as a limitation in energy models to adequately reflect decision-making behavior may in large part be a reflection of the lack of sufficient generalizable evidence in the empirical literature on heating equipment adoption, specifically in how households incorporate known factors such as cost, awareness, comfort and reliability in different contexts. Models can at least separate the two stages in adoption of deciding to invest in new equipment from technology selection and reflect differences in perceived affordability across income groups.

The rest of the article is organized as follows. In the next section we review and synthesize knowledge about decision-making around EHP adoption. Following that, we review how existing energy models project EHP adoption in energy/climate futures. In the discussion section, we combine insights from previous sections to make recommendations for deeper integration between social sciences and energy models, including needed advancements in both field research and modeling.

## State of knowledge about drivers of EHP adoption

### Literature search methodology

Heat pumps are a relatively new technology, because of which the literature on their adoption is sparse. In order to systematically research this literature, we used the following search criteria in Scopus, Web of Science, and Google Scholar to identify peer-reviewed journal articles in English. We combined variations of keywords for “heat pump” and “adoption”. Variations of “heat pumps” included “heat pump systems,” “heating,” “geothermal heat pumps,” “ground-sourced heat pumps,” “air source heat pumps,” and “heating system.” Note that in Europe heat pumps are often covered under a category called “renewable heating systems,” which may include other renewable fuels such as biomass pellets. The search yielded 212 articles, with a considerable skewness in vintage toward the present. Manual perusal of these articles yielded only 42 articles that sought empirical insights into adoption drivers. Most of the rest were engineering, physics or modeling analyses of various configurations of heat pumps to assess performance and/or costs. The empirical studies were of two types: field research, some as part of ABMs studies; and statistical analyses of large-N, typically nationally representative, surveys. These two strands have their respective limitations. The former provide useful insights on drivers of EHP acquisition, but for relatively small geographic areas with limited, if any, external validity. The second type of empirical studies, the statistical analyses of surveys, have the advantage of a large sample size, but because they have access to ownership patterns rather than acquisition decisions, they cannot offer insights on behavior. More than two-thirds of the studies were based in Europe. One notable exception is a study by Antonopoulos et al., wherein they examine decision-making in a survey of 10,000 American households that have undertaken any kind of technology adoption.^5^ Otherwise, most studies based on field research are mostly from Northern Europe, whose applicability to the US is limited due to differences in the types of heating equipment, house construction and homeowners’ values and norms.

We synthesize some of the findings from both types of studies. At the outset, it is noteworthy that decisions involve two stages, in which different factors may come to bear: in the first phase, households decide to replace their heating equipment; and in the selection phase, households choose among available technologies/systems. Each phase may involve different decision criteria. Policy incentives could accelerate premature retirements and make EHP more attractive among available options. To predict adoption rates at a national scale, one would need to know *which* factors come to bear in each stage, *how* households incorporate these factors in their decision rules, and what contextual and socioeconomic factors influence how these decision processes vary across a population.

### Current state of knowledge on EHP adoption

In the papers reviewed, the conditions that cause households to enter the first stage decision—considering replacement—are not well understood. As mentioned, heating equipment typically lasts over twenty years. However, the useful life is use-dependent and subjective, since people may have different preferences for balancing risks of breakdown against undertaking major upgrades. Households that are satisfied with their heating equipment are unlikely to change their equipment.^14^ Antonopoulos et al.’s study indicates that equipment breakdowns were one of the many motivations for adopting new home technologies in the US.^5^ It is curious, given the high prevalence of central air distribution systems with heating and cooling in the US, whether failures in air conditioning (AC), and not just heating, equipment, contribute to triggering heat pump adoption.

In Europe, a few studies that examined the causes of equipment changes find a broader set of motivations. Households typically confront a problem, identify a new opportunity, or move to a new home, as an impetus to switch their heating system.^15^ Curtis et al. found in a representative survey of Ireland that fuel costs and heating equipment “not working well” were more common reasons than equipment failing for their replacement.^16^ In Sweden, a marketing campaign by a municipal utility convinced over three-quarters of over 700 residents with electric resistance heat who expressed no prior need for a new system to adopt service from a biomass-based district heating system.^17^ In a small (6) sample of existing and new home owners in New Zealand, information was insufficient to overcome inertia to adopt energy-efficient hot water systems.^18^ Lillemo et al. found that reducing operating costs, improving air quality and replacing broken equipment were the primary reasons to change equipment in Norway.^19^ No other evidence was found on what additional incentives motivate change, other than moving to a new home.

In the second stage decision—when households do decide to replace old heating equipment—they tend to be creatures of habit and retain the prior fuel and technology.^20^ A few review articles and more recent studies focusing on Northern Europe provide evidence for the influence of several factors in shifting to EHP. Overall, cost considerations are the most widespread, though the components of cost that households consider—between initial cost, running costs or financing—differs across studies. Operating costs of heating tend to trigger a search for new equipment, but the latter’s upfront costs is an adoption barrier. Otherwise, while some studies corroborate each other on factors such as comfort, reliability, pro-environment attitudes and appearance, none of them are as common as cost. We next review studies that survey actual adopters, and then those that investigate hypothetical preferences, and then address the intention-action gap.

Heiskanen and Matschoss^9^ review differences in adoption rates across European countries and residential renewable energy technologies, of which EHPs are one category. For EHPs, they find that affordability is a primary driver of adoption. Younger, more educated, and wealthier people have a higher likelihood of adopting new innovative products, which would matter for EHP if they are perceived as such. In their review of EHP markets, Gaur et al.^10^ identify market barriers, such as policy and regulatory uncertainty, public acceptance, and economic factors. Their review suggests that besides cost the lack of familiarity with EHP technology can inhibit uptake. But these reviews do not illuminate how lifestyles of different demographic groups influence their decision process nor what type of knowledge households would want and how they would incorporate it.

Among more recent country studies in Europe, comfort^20^ or a desire for less fossil fuel use^20^^,^^21^^,^^22^ seem to influence the choice of an EHP over other heating equipment. Furthermore, in Germany two studies find that educated households are sometimes observed to put more emphasis on adopting a proven (i.e., reliable) heating system—choosing gas over EHP.^23^^,^^24^ A study in Sweden finds annual heating costs to be the highest priority among a majority of households, followed by system reliability.^14^ Notably, environmental attitude, including toward climate change, was a low priority in that survey. Respondents in this study ranked EHPs the highest among alternatives for having relatively more advantages. In Italy, a study on the propensity to adopt biomass pellet heating systems found that households segmented along their propensity to adopt new technologies, among other factors such as their perception of heating system characteristics.^25^ However, this study may not reveal other decision criteria because it was designed to test Rogers’ theory of diffusion, which focuses on people’s receptiveness to new, innovative products.

Several studies investigate people’s hypothetical willingness to adopt EHPs. Jingchao et al. use stated preference surveys to determine what influences people’s willingness to pay (WTP) for EHPs in China. They find that being female, having science literacy and local environmental concerns increase WTP.^26^ Côté and de Brauwer show that Germans would lease EHPs to avoid the technical risks of a new technology.^27^ Corbett et al. find that policy awareness is a strong determinant of people’s willingness to adopt EHPs in Canada.^28^

Results from stated preference studies are indicative of only people’s inclination, that is their willingness to *consider* EHP. Their intentions may depart from these inclinations, and furthermore their actions may not be consistent with their intentions—the latter being the well-known intention-action gap.^5^^,^^29^ Thus, there are likely to be additional barriers that inhibit those inclined, in principle, to adopt EHPs. Many studies find that the lack of knowledge about the technology and its true installed cost are significant barriers to adoption.^20^^,^^29^^,^^30^^,^^31^ One study in Germany finds that home owners in search of new equipment scrutinize EHPs costs more closely than do those of familiar technologies.^20^ In the Netherlands, a survey found a distinct difference between households’ willingness to adopt and the willingness to put in effort toward adoption.^29^ Further, in this study many respondents felt they lacked the knowledge, financial means or time to adopt EHP despite having a positive attitude toward them. Karytsas et al. find that households WTP is well below installation costs, and that payback periods even for ground-sourced EHPs, which have lower operating costs than conventional EHPs, are not acceptable for the majority of respondents in three European countries.^32^ It seems people who express an inclination to adopt EHP underestimate practicalities that they would consider when actually deciding.

One potential barrier that is rarely discussed in the US context is homes’ physical “readiness” to switch to EHPs without modifications, either to the heat distribution systems or to overall insulation. In the US, where air-to-air heat pumps (ASHPs) are the norm, homes with baseboard heaters, which are common in the Northeast, would need to put in duct systems or more complicated configurations of heat pumps. Households may also require electrical upgrades to support the power use by EHPs. The additional costs are context-specific, and hence not known *a priori*, but they certainly would exacerbate the affordability barrier. A recent study in the UK^33^ shows that many homes would require costly upgrades, either to improve insulation or resize distribution systems to accommodate EHPs. Over half the households surveyed in that study were not willing to pay for such upgrades. This study also reinforces the importance of prior knowledge about EHP, which may be a prerequisite to considering adoption. Another UK study identifies the lack of trained contractors and complex user experiences among EHP owners as contributors to the slow adoption of EHPs in the UK.^34^ A few studies in Europe^16^^,^^20^ also found that owners of smaller homes are unable to adopt EHP or other renewables due to space constraints, whereas bigger houses have the required space for installing EHP as the primary or secondary backup heating system.

Statistical examinations of large cross-sectional household surveys provide insights on socioeconomic characteristics that correlate with whether households *have* EHP. Since they do not have information on adoption decisions, they cannot distinguish first stage decision factors from fuel/technology selection criteria. As such, they provide only indirect proxies for decision criteria. Many studies show that moderate climate and lower electricity rates are associated with higher EHP ownership rates in the US.^6^^,^^35^^,^^36^ Shen et al. show that rebates on EHP in North Carolina in the US may have incentivized households to adopt EHP.^37^ Poblete-Cazenave and Rao show that having better insulation, being in urban areas and younger, also increase the likelihood of adoption.^6^ Some of these factors directly enter the economic calculus, such as moderate climate (due to higher EHP efficiencies in operation), lower electricity prices and better insulation (which reduce the required capacity and related upfront capital cost). However, age and urbanity are likely proxies for unobserved decision criteria. A recent study based on a survey in Vermont finds spatial clustering of EHP owners, which may be indicative of a peer-effect.^36^

In summary, previous research sheds light on *factors* that are considered in decision-making or on household characteristics that increase the likelihood of EHP ownership. However, we do not gain from these studies a complete picture of *decision-making processes.* For instance, cost factors, particularly related to upfront installation, seem the most widespread. However, none of the studies elicits the calculus used to make decisions. Only a few studies that ask about WTP make mention of acceptable payback periods. However, as noted earlier, stated preferences may depart from actual behavior. All types of costs may not enter into decisions,^20^ and those that do may be considered alongside other non-economic factors. Knowledge about EHPs seems to be a common barrier. However, it is hard to know whether because of ignorance EHPs were simply left out of the choice set, or if the lack of familiarity with the technology caused a cognitive bias against EHP (such as, perceiving them as less reliable than they are). Other non-cost factors that were identified in a few studies include perceived comfort, system reliability, familiarity, and attitudes toward the environment and innovation, among others.

Without knowing how these factors are prioritized, weighted, or conditioned by circumstance, it is hard to estimate, let alone project, their influence. Even if one were to define heuristics for decisions, even selecting the most salient factors seems difficult. Aside from cost and awareness, most of these factors are identified in a subset of studies, each having a different sample of households in different cultural and economic environments, which may have different economic, social and knowledge endowments.

### Relevance of other household technologies to heat pumps

To understand EHP adoption decisions better, one could cast a wider net beyond heating systems to the literature on residential energy technologies in general, including solar rooftop PV (SPV), or to the voluminous literature on energy efficiency technologies or energy conservation measures (ECMs).^38^^,^^39^^,^^40^ They all share some common characteristics, such as involving new potentially unfamiliar technologies with high upfront costs that may be driven by potentially complicated government incentives. As such, households may have similar barriers to embracing them stemming from these characteristics.

However, there are other distinct differences between these technologies and heating systems, which may lead households to apply different decision rules. SPV are external. As such, they do not alter indoor living conditions (e.g., comfort). EHP is largely internal equipment, which may not carry status value as much as do SPVs. Further, EHP may involve intrusive changes to heat distribution systems because they typically involve lower temperatures of heat circulation compared to fossil fuels, and they may improve indoor air quality. Installing SPV, in contrast with EHP, does not entail a significant change in environmental conditions (e.g., pollution) or lifestyle (e.g., EVs and driving behavior).

ECM such as insulation or windows may share further similarities with EHP because their installation affects comfort, and has other side effects, such as changing home aesthetics. However, these impacts are largely known upon installation. In contrast, heating systems involve potentially unknown ongoing maintenance and varying costs from weather-dependent performance.

Given these differences, decision factors concerning SPV or ECM are likely to differ from those of EHP. For example, status signaling is more likely to influence SPV adoption than that of EHP or even ECM. On the other hand, indoor air quality would likely influence EHP adoption, if at all, but not SPV or ECM. Thus, applying models of decision making from SPV of ECM could be misleading. Furthermore, the scope of this review would increase to a prohibitive extent. To be clear, this doesn’t rule out that some of that literature may be applicable to EHP adoption, or that this review in turn may also shed light on the adoption of other technologies in the home. We leave such investigation for future research.

Aside from empirical studies, scholars have drawn on various theories to describe heating equipment adoption behavior, arising from the fields of economics, sociology and psychology (for a synthesis, see studies by Wilson and Dowlatabadi^41^ and Frederiks et al.^42^). These theories hypothesize how decision-making is influenced by awareness (e.g., about new technologies and risks), attitudes and intentions (e.g., toward innovation and environment), perceptions of self-efficacy or social conformity, consequence (e.g., private economic costs and health), and other external constraints (e.g., income or physical home conditions). The factors discussed earlier have been shown to play a role, but their strengths vary among studies. Population characteristics or market conditions between studies are not examined with the purpose of determining why decision factors might vary. Furthermore, the ordering of causality of relationships described by the theory is infrequently validated.

Models require valid decision rules, including the strength of different factors when several of them influence decisions. Given the heterogeneity in how people weigh different factors, one would need to know how these weights vary with population characteristics. The aforementioned theories have backing evidence, but not to such a degree. Some ABMs have assumed that household decisions can be represented with the Theory of Planned Behavior (TPB). They use surveys to estimate weights for different decision factors. However, as discussed later, these studies do not validate these theories by testing the fit of their estimates against other theories. They remain largely hypothetical scenarios of outcomes under the assumption of such decision-making behavior.

The aforementioned synthesis reveals the challenge of deriving generalizable decision rules to characterize EHP adoption decisions. There has been little, if any, investigation in the US of the first decision stage of why household enter the market for a new heating system in the first place. From the European studies and one US study one learns that price shocks, equipment failures or moving to a new home are common motivations. With regard to technology selection criteria, the evidence shows that households vary in their knowledge of EHPs. Those familiar enough with EHPs value installation costs and some subset of other factors that vary across populations, such as comfort, reliability and environmental impact. Given the qualitative nature of these findings, it is unclear how to simulate a decision process, even just based on cost, and how to differentiate households into groups with like behavior. In order to assess how to better apply the state of knowledge to inform household energy models, we first review how various types of models have represented household decisions.

## How household energy models represent heating equipment adoption decisions

Energy models are typically grouped into three categories, integrated assessment models (IAMs), energy system models (ESMs), and ABMs.^12^ The purpose of modeling household heating equipment adoption decisions is to project future household energy demand, which could in turn feed into energy supply cost optimization scenarios, economic production functions or simulations of future energy system behavior, all of which may be modeled at different scales depending on the model purpose. See refs.^12^^,^^43^ for a more detailed overview of energy-economy models in climate research. At the most aggregate level, IAMs and some ESMs do not model households at all. Instead, they derive market shares for different technology/fuel combination in the residential sector using mathematical functions that include technologies’ life cycle costs. We call these Market allocation Models (MAMs). Prominent examples in the US include NEMS^44^ and GCAM-US,^7^ while global IAM examples include IMAGE^45^ and TIMES. Then there are a subset of ESMs that are detailed (“bottom-up”) building simulation models, typically from the architecture and building engineering community, which model physical building characteristics to simulate heating energy demand—we call these building models (BMs).^11^ Usually BMs focus more on heating equipment operation than on adoption, but a subset that are soft-linked to or part of IAMs project future operation (for e.g., STURM^46^ in MESSAGE, or Res-IF in IMACLIM^47^), which requires assumption on future adoption as well. The most well-known example in the US is ResStock, which has been used to assess the attractiveness of EHPs across different building types.^48^ Lastly, we review ABMs, whose raison d’etre is to model behavioral rules in households involving interactions among them, such that markets’ emergent properties can be observed through simulation.^49^

In the following, we crystallize the best practices from these models in terms of how they reflect the evidence in the reviewed literature, rather than comprehensively summarizing all models. We will draw examples from three MAMs (NEMS, GCAM-US, and IMAGE), and one BM (ResStock). We present the two decision stages and then discuss best practices in ABMs separately.

### First-stage decision

Among the reviewed MAMs, only NEMS models a two-stage decision, whereby the market for replacement is first determined before determining fuel and technology choice. The model determines the size of the replacement market based on the equipment stock in the base year and a retirement rate for each equipment type derived from an assumed useful life. The market for new technologies is further constrained to a (seemingly arbitrary) value of 20 percent of retirements among single-family homes to reflect the bias in the population toward retaining their previous fuel/technology. The second stage decision rule to determine the replacement fuel/technology is applied only to this 20 percent.

### Fuel/technology choice

MAMs have market allocation rules for different technologies based almost exclusively on life cycle costs, which include upfront and operating costs. In order to prevent unrealistic “winner-take-all” behavior—where the most cost-efficient technology would be adopted by all households in the market—models employ different mechanisms to constrain the market shares of new entrants.^50^ Typically, these are bias parameters or weights that are calibrated using survey data to baseline market shares. As such, they are “knobs” that proxy for, rather than explicitly represent, the range of different underlying market dynamics that cause gradual market takeover (e.g., fragmented markets).

Methodologically, heating technologies’ market shares are derived using multinomial logit model estimations, which assume these technologies are perfect substitutes. The shares represent the probability of a household adopting a particular technology conditional on the economic costs of the technology and an “intangible” preference factor, since the costs alone would not correctly predict existing market shares. The coefficient of the cost variable defines the sensitivity of the market shares to changes in cost. Symbolically, they calibrate an equation of the form in the following, for *j* fuel/technology combinations, where *LCC* is its life cycle cost, *c* may be the cost sensitivity coefficient derived from a multinomial logit, *b or a* are “knobs” used by the modeler to prevent winner-take-all behavior, which may also be calibrated from survey data.

(Equation 1) $$\text{Shares}=\frac{a.e^{\left(b+c{LCC}_{j}\right)}}{\sum_{\forall j}a.e^{\left(b+c{LCC}_{j}\right)}}$$

This form allows the models to project future market shares based on changes in technology cost and physical household conditions that may influence the heating operating costs. This is how heterogeneity in the building stock can be included, as with NEMS or ResStock. This cost coefficient also allows for policies that change costs, such as rebates for EHPs, to influence projections. However, the preference/bias parameter is typically a static residual factor that represents unexplained market conditions in the base year. As such, changes in customer preferences over time would require a change to these bias parameters. This would be important, because with growing market shares of new technologies, peer effects, growing awareness or other technology spillovers may well increase customer receptiveness to EHPs. However, the present literature lacks an empirical basis to calibrate future values for such a parameter.

BMs that integrate with IAMs, such as STURM^46^ (with MESSAGE) or Res-IRF^47^ (in IMACLIM-R), use a variation of the aforementioned approach (Equation 1) of allocating technology shares to households based on LCC. Rather than using a single parameter to control a technology’s market share, they include a technology-specific time-varying “intangible cost” function that includes a parameter for transaction costs and a countervailing positive time-sensitive technology spillover that represents market maturation. As such, the influence of this intangible reduces with growing market share. However, all households still implicitly have the same cost-minimizing decision rule. Some BMs, in contrast to MAMs, model some household heterogeneity rather than a single residential sector, and therefore have the potential to incorporate different decision rules. However, most models limit this functionality to modeling differences in home physical characteristics in order to more accurately represent heating operation. For example, ResStock creates a synthetic dataset of over 500,000 households based on simulations of surveyed households in order to represent the heterogeneity in building shells across the US.^48^ However, other than income, the database has few descriptors that can enable differentiation in household behavior.

BMs that project energy demand into the future typically have a cohort model that simulates retirement and new construction, and a model for technology adoption, which typically assume standard economic payback or net-present-values.^48^ One BM, TIMES,^51^ stands out for differentiating adoption decisions by household group, by assigning them different discount rates (or rates of return on their investment) and differential access to new technologies. Such BMs could be used to investigate different heuristics for triggering premature retirement. For instance, a study of the Dutch building stock shows that a younger building stock discourages renovations due to the shortened payback horizon.^52^

In summary, if one were to draw out the features from all the state-of-the-art IAM/ESM models that best represent demand behavior, they would include: a two-stage process that first identifies a subset of households that are in the market for a new heating system; further subdivision of the households in the market by geography and income; and the assignment to each subgroup of different building shell efficiencies, climate conditions, discount rates, and access to new heating technologies. However, it is notable that other than the use of income-dependent discount rates, such as in TIMES, no other behavioral attributes can be found in the reviewed models. We will come back to this in the discussion section where we discuss potential model improvements, including the adoption of best practices across the field, as well as further refinements that could push the envelope further by incorporating insights from the social science literature.

## Behavioral research with ABMs

ABMs model consumer energy choices, including behavioral drivers of technology adoption (see Table 1). The ABMs that model adoption choices in our review have in common is that they model some form of interaction between households and examine emergent properties from these interactions. ABMs are typically of smaller geographic scale than MAMs or ESMs, which allows them to incorporate greater heterogeneity in household behavior. For instance, some households may repeat prior technology choices, other may imitate peers, and only a subset may review costs. Some studies aim to develop household archetypes based on some combination of behavioral, physical and socioeconomic attributes.^58^^,^^59^

**Table 1:** Summary of key features and insights from ABMs

Study	Unit of analysis^a^	Behavioral influences	Utility function assumed (if any)	Empirical basis for model	Validation method	Interaction variables	Emergent property (outcome)
Snape et al.,^53^	ASHP, GSHP	Economic payback; peer effect; technology hassle factor	Weighted sum of three factors: $X_{decision=}w_{econ}.x_{econ}+w_{social}.x_{social}-w_{hassle}.x_{hassle}$	Secondary sources	None	Peer influence	1. Adoption plateaus in three years 2. Peer effect only with 15% of neighbors
Niamir et al.^54^^,^^55^	Fuel switch	Norm activation theory (NAT)—considering knowledge K-> motivation M-> consideration C -> action	Expected utility = share of income to be spend on composite good ∗ (total budget – energy cost) + share of income to be spend on energy appliances ∗ energy cost	800 households in Navarre, a province in Northern Spain.	None	Adjust awareness and motivation factors to be the mean of 8 closest neighbors.	1. Bottom income groups (<10k, 10–30, 30–50) are most likely to switch to low-carbon technologies. 2. Positive psychological influence reduces energy use by 67%. 3. Exchanging knowledge lead to 78.25% decrease in energy use
Sopha et al.^56^^,^^57^	Three heating system—direct electric heating, individual wood-pellet stove, and air-to-air heat pump.	Theory of planned behavior (TPB)—repetition, deliberation, imitation, social comparison.	U = (c1∗ Attitude to heating system adoption + c2 ∗ perceived behavioral control + c3∗personal norm) ∗ (1 – c4∗social influence) + (number of adopters∗ social influence co-efficient)	270 Norwegian households	Network topology and historical data for wood-pallet adoption rate is adopted for validation.	Social interaction is defined by spatial proximity.	1. Heat pump adoption rate is higher for all regions 2. Electricity price fluctuations encourage wood pallet adoption.
Lee et al.^58^	Solar and photovoltaic systems, heat pump	Multiple attribute decision making method (MLR)	U = $\sum_{j=1}^{n}w_{j}v_{j}\left(x_{ij}\right),i=1,2,3,..,40$	Stock model for 7790 owner-occupied dwellings in the UK	Historic installation rates for loft insulation and cavity wall insulation for 1996–2008	Recommendation from neighbors (although no values were provided), no description.	1. Subsidies did not influence energy reduction
J Sachs et al.^59^	Gas boiler, heat pump	Linear optimization-based market analysis based on supply-demand elasticities	NA	A hypothetical region	NA	Number of adopters in the whole market	Heat pump adoption rate is greater due to low fuel cost in comparison to other options.
Meles & Ryan^60^^,^^61^	Home heating system—two generic options are given	Theory of planned behavior, ordered probit model, logit model	U = $w_{econ}*U_{econ}+w_{psychology}*\;U_{psychology}+w_{network}*\;U_{network}$	1,208 households in Ireland	NA	Percentage of adopters in the market	1. High upfronts costs (rebates) limit (encourage) heat pump installation 2. Education, number of bedrooms related to higher heat pump adoption

a Heat pump, heating equipment, energy efficiency, home improvement investment in general, other. ASHP, air-source heat pump; GSHP: ground-source heat pump.

While studies may differ in the types of emergent properties they aim to examine, they usually assume a particular behavioral theory or set of influences that drive their emergent properties. The TPB is the most common, wherein several psychological factors, such as attitudes and perceptions of agency, influence intention. Authors usually construct the decision rule as a linear utility function with some combination of economic, psychological, and social drivers, weighted by their relative influence (Equation 2 as follows).

(Equation 2) $${Utility}_{i}={w_{eco}.Eco}_{i}+{w_{psy}.Psych}_{i}+{w_{soc}.Soc}_{i}$$

The economic payback function is typically more nuanced in ABMs compared to MAMs and BMs by virtue of including more detail in households’ energy costs such as differentiated taxes/subsidies, discount rates or operating costs. Psychological factors may include knowledge/awareness, attitudes (toward the environment, technology, and effort involved), and agency/control. Social influences usually are modeled as a neighborhood or peer effect. Often ABMs use bespoke surveys to uncover the presence and strength of these factors, which standard national surveys do not contain. The survey data are typically used to calibrate parameters in their decision rules. One advantage of such a rich representation of decisions is that ABMs can include other agents, such as government, contractors or housing associations.^62^

ABMs provide useful insights on the importance of behavioral attributes. For instance, one study in the UK show that a more diverse set of heating technologies may be adopted under assumptions of household heterogeneity relative to a homogeneous population.^59^ A study in Norway illustrates that the relative competitiveness of biomass pellets relative to EHP depends on multiple attributes, including price, performance and environmental quality.^56^ Another study in the UK simulates how a “hassle” factor associating with installing EHPs can cause a tipping point, which may explain significantly lower adoption rates in UK relative to the rest of Europe.^53^

As with other types of models, limitations of ABMs are the validation of the underlying relationships. Surveys are often done with the largest feasible population, which may nevertheless be unrepresentative of a greater population. Most ABMs simulating EHP adoption have been developed for European populations, so their translation to the US market is questionable. In some cases, the underlying relationship is based entirely on assumption, for the purpose of illustrating how differentiating behavior in a population could lead to unexpected outcomes.

## Discussion

Currently most national models represent heating equipment technology market shares rather than household decisions. Here we synthesize some constructive suggestions for how energy models can enhance realism in their EHP demand forecasts (See Table 2). We discuss these in two categories: incremental improvements that can be informed by existing models and literature and available data; and deeper structural model development that would have to be built on new data collection. In both, we address, where possible, three aspects of the adoption decision process: the first stage decision to replace heating equipment; the technology selection process; and household heterogeneity.

**Table 2:** Key model features in the literature and suggested future improvements

	Common practice	Best practices in reviewed models	Incremental improvement	Long-term improvements
*Household heterogeneity*	None	Differentiated by income (e.g., GCAM), geography and building type (e.g., ResStock)	Combined best practices, calibrated to national survey data	Identify behavior-differentiating dimensions; Include differential influence of social interaction
*First-stage decision:* Equipment replacement	None	Assumed retirement rate (e.g., NEMS)	Include cohorts (e.g., by equipment age distribution)	Stochastic triggers (e.g., price shocks) based on data
*Second-stage decision:* Technology choice				
Technology learning	Status quo bias (calibrated to base year data)	Allow market maturation, stylized (e.g., TIMES)	Adopt best practices	Parameterize technology know-how, consumer perceptions
Life cycle cost (LCC)	Average LCC drives adoption rates	Income-differentiated switching cost (e.g., ResIRF/IMACLIM)	Income-differentiated discount rates, intangible costs	Differentiated cost functions based on group-specific constraints, internal retrofit costs (electrical, distribution)
Social/government interaction	None	ABMs, represented but not validated	–	Survey-driven peer-effects, utility/government awareness programs

Notes: Long-term, unlike Incremental, improvements, require additional data collection.

### Incremental model improvements

We have emphasized the importance of the retirement rate for heating equipment. Explicitly incorporating the retirement decision may be the most important addition to energy models, because it is a gating factor for EHP adoption, and because there are feasible empirically based modeling strategies. NEMS’s replacement market is an example of this first phase. Data on the age distribution of heating equipment in energy surveys (for e.g., the Annual Housing Survey or Residential Energy Consumption Survey), annual sales, or building permits can serve as starting points for estimating retirement rates. Additional retirements can be triggered by hazard functions that simulate equipment failures or fuel price shocks.

The next stage of technology selection involves a strong status quo bias, which must be included in a dynamic form to allow for the influence of future policies and market maturation on households’ awareness of EHP. A study on electric vehicle adoption includes owners’ existing vehicle with proxies for switching costs^63^ to model the status quo bias. As EHPs get more widespread and known, either through social networks or policy efforts, this bias may reduce. Models could include an additional technology-specific parameter to counter this bias parameter that reflects market maturity and grows with time, akin to the time-dependent countervailing technology spillover in Res-IRF.

Third, differentiating households at least by income and geography is essential for reflecting different costs and perceptions of affordability. Although most models select technologies based on life cycle costs, our review does not find sufficient evidence that households account for costs on that basis. Upfront costs may dominate cost perceptions. Nevertheless, in the absence of clear evidence on how households factor economics in their decision, modeling payback periods or life cycle costs seems reasonable. In WTP studies households respond to questions of affordability framed in payback terms, giving the impression that they do understand life cycle costs. However, they could just be taking the lead of researchers from their question design. Modeling life cycle costs also offers the flexibility to model different kinds of household constraints and policy support, such as rebates on installed costs verses reduced electricity rates. Thus, modeling income groups with differentiated discount rates and payback period hurdles is state-of-the-art. Some studies calculate the equivalent of an elasticity of WTP to income, which can be implemented with income-differentiated groups.

### Deeper integration between models and empirical research

The two-stage decision can be better modeled with a deeper understanding of market dynamics and household conditions. We have learned that price shocks, moving house and aggressive marketing (in Europe) have all led to premature retirement of heating equipment. Future fieldwork that targets EHP adopters and focuses on uncovering the tipping point for these decisions would be useful. Some triggers (e.g., price shocks and equipment failures) can be represented stochastically, informed by historical data.

Market maturation and the influence of policy incentives need to be understood to model realistic bias parameters for technologies. ABMs have an important role here, since they focus on modeling interaction between agents. They can study knowledge spillover among households, or government influence on households with different trust levels. However, future ABMs need methodological improvements to ground them in reality. We have pointed out issues with validation and generalizability previously. The best practices for developing ABMs have been reviewed elsewhere.^49^

Regarding technology choice, ideally models would be derived from empirically validated theories of technology selection that identify factors that people consider, their relative strengths, how they combine to influence decisions, and how all these elements vary across the population. From our review, we have learned about the factors that seem to enter people’s calculus, and that the set of factors and their relative importance vary for different population groups. However, the relative strengths of these factors and how they vary across the population has not been quantified.

We need further empirical research to determine the weights of these factors for a representative sample of the population, so that the population can be clustered into groups with similar behavior. The most important behavioral attributes that differentiate choices need to be drawn out and represented. Since there is an intention-action gap, surveys need to target households that have been in the market for new heating equipment. Reports of surveyed attitudes should state clearly in which phase the features are observed; for example, if they reflect intention rather than adoption. With so many gaps in understanding, the likely variation in how people make decisions, and the range of circumstances in which they make them, we need national-scale surveys that can yield generalizable results.

Other than affordability, the literature offers little guidance on how to differentiate behavior across the US population. Even with regard to affordability, the literature his little guidance on understanding affordability constraints in low-income communities, such as upfront cash constraints or credit eligibility. One can conjecture many reasonable criteria for differentiation, as have been modeled in ABMs, such as spatially sensitive peer effects, differences in knowledge about EHPs between urban and rural households or between different education levels, or receptivity to new innovative technologies by wealthier and younger households. However, these effects are known qualitatively. The strength of their influence relative to each other for different population groups has yet to be quantified. Iteration could be undertaken between ABM sensitivity studies and rapid polling techniques that probe factors determined to be particularly important for overall transition rates or the distribution of benefits. Finally, there are other factors that may play a role that have not been investigated. As mentioned earlier, the need to replace AC equipment, or the side benefit of cooling in temperate regions that are increasingly experiencing extreme heat, merits investigation. Landlords and single-family homeowners may have different sets of incentives and decision criteria. Among new home purchasers, commercial entities that purchase for resale may be more inclined to replace heating equipment. Contractors are an important intermediary who influence households’ knowledge and behavior, and whose own knowledge and training on and incentives to sell heat pumps likely vary widely and merits investigation. Changes in government regulations or outright bans of fossil fuel-based heating equipment increase the attractiveness of EHPs, but their enforcement may be important in communities that distrust government.

## Acknowledgments

N.D.R. and T.C.B. were partly funded by a 10.13039/100000001National Science Foundation Grant on Growing Convergence Research, awards 0008428 and 2219255, respectively.

## Author contributions

Conceptualization: N.D.R.; investigation: N.D.R. and M.R.K.S.; writing – original draft: N.D.R.; writing – reviewing and editing: T.C.B. and M.R.K.S.

## Declaration of interests

The authors declare no competing interests.

## References

[1] Intergovernmental Panel On Climate Change (Ipcc) (2023). Climate Change 2022 - Mitigation of Climate Change.

[2] United States Department of State and United States Executive Office of the President (2021).

[3] Lutz J.D., Hopkins A., Letschert V., Franco V.H., Sturges A. (2011).

[4] Michael Thomas (2024). https://www.distilled.earth/p/heat-pumps-outsold-gas-furnaces-in?utm_campaign=email-half-post&r=aho53&utm_source=substack&utm_medium=email.

[5] Antonopoulos C.A., Fuentes T.L., McCord K.H., Rackley A.L., Biswas S. (2024). Regional assessment of household energy decision-making and technology adoption in the United States. Energy Pol..

[6] Poblete-Cazenave M., Rao N.D. (2023). Social and contextual determinants of heat pump adoption in the US: Implications for subsidy policy design. Energy Res. Social Sci..

[7] Sampedro J., Iyer G., Msangi S., Waldhoff S., Hejazi M., Edmonds J.A. (2022). Implications of different income distributions for future residential energy demand in the U.S. Environ. Res. Lett..

[8] Chadwick K., Russell-Bennett R., Biddle N. (2022). The role of human influences on adoption and rejection of energy technology: A systematised critical review of the literature on household energy transitions. Energy Res. Social Sci..

[9] Heiskanen E., Matschoss K. (2017). Understanding the uneven diffusion of building-scale renewable energy systems: A review of household, local and country level factors in diverse European countries. Renew. Sustain. Energy Rev..

[10] Gaur A.S., Fitiwi D.Z., Curtis J. (2021). Heat pumps and our low-carbon future: A comprehensive review. Energy Res. Social Sci..

[11] Mastrucci A., Niamir L., Boza-Kiss B., Bento N., Wiedenhofer D., Streeck J., Pachauri S., Wilson C., Chatterjee S., Creutzig F. (2023). Modeling Low Energy Demand Futures for Buildings: Current State and Research Needs. Annu. Rev. Environ. Resour..

[12] Krumm A., Süsser D., Blechinger P. (2022). Modelling social aspects of the energy transition: What is the current representation of social factors in energy models?. Energy.

[13] Strazzera E., Meleddu D., Contu D., Fornara F. (2024). Willingness to pay for innovative heating/cooling systems: A comprehensive appraisal of drivers and barriers to adoption in Ireland and Italy. Renew. Sustain. Energy Rev..

[14] Mahapatra K., Gustavsson L. (2010). Adoption of innovative heating systems—needs and attitudes of Swedish homeowners. Energy Efficiency.

[15] Hecher M., Hatzl S., Knoeri C., Posch A. (2017). The trigger matters: The decision-making process for heating systems in the residential building sector. Energy Pol..

[16] Curtis J., McCoy D., Aravena C. (2018). Heating system upgrades: The role of knowledge, socio-demographics, building attributes and energy infrastructure. Energy Pol..

[17] Mahapatra K., Gustavsson L. (2009). Influencing Swedish homeowners to adopt district heating system. Appl. Energy.

[18] Grieve C., Lawson R., Henry J. (2012). Understanding the non-adoption of energy efficient hot water systems in New Zealand. Energy Pol..

[19] Lillemo S.C., Alfnes F., Halvorsen B., Wik M. (2013). Households’ heating investments: The effect of motives and attitudes on choice of equipment. Biomass Bioenergy.

[20] Michelsen C.C., Madlener R. (2012). Homeowners’ preferences for adopting innovative residential heating systems: A discrete choice analysis for Germany. Energy Econ..

[21] Jung N., Moula M.E., Fang T., Hamdy M., Lahdelma R. (2016). Social acceptance of renewable energy technologies for buildings in the Helsinki Metropolitan Area of Finland. Renew. Energy.

[22] Psarra I., Turhan E., Ghassemialiabadi F. (2024). Zero carbon, some nuisance: Exploring the viewpoints of heat pump owners and their neighbors in Groningen, Netherlands. Energy Sources B Energy Econ. Plann..

[23] Michelsen C.C., Madlener R. (2016). Switching from fossil fuel to renewables in residential heating systems: An empirical study of homeowners’ decisions in Germany. Energy Pol..

[24] Braun F.G. (2010). Determinants of households’ space heating type: A discrete choice analysis for German households. Energy Pol..

[25] Franceschinis C., Thiene M., Scarpa R., Rose J., Moretto M., Cavalli R. (2017). Adoption of renewable heating systems: An empirical test of the diffusion of innovation theory. Energy.

[26] Jingchao Z., Kotani K., Saijo T. (2018). Public acceptance of environmentally friendly heating in Beijing: A case of a low temperature air source heat pump. Energy Pol..

[27] Côté E., Pons-Seres de Brauwer C. (2023). Preferences of homeowners for heat-pump leasing: Evidence from a choice experiment in France, Germany, and Switzerland. Energy Pol..

[28] Corbett M., Rhodes E., Pardy A., Long Z. (2023). Pumping up adoption: The role of policy awareness in explaining willingness to adopt heat pumps in Canada. Energy Res. Social Sci..

[29] Okur Ö., Fiori F., Fouladvand J. (2024). Adoption of renewable heating systems and thermal energy communities in the Netherlands: An empirical study. Energy Rep..

[30] Peñaloza D., Mata É., Fransson N., Fridén H., Samperio Á., Quijano A., Cuneo A. (2022). Social and market acceptance of photovoltaic panels and heat pumps in Europe: A literature review and survey. Renew. Sustain. Energy Rev..

[31] Karytsas S. (2018). An empirical analysis on awareness and intention adoption of residential ground source heat pump systems in Greece. Energy Pol..

[32] Karytsas S., Polyzou O., Karytsas C. (2019). Factors affecting willingness to adopt and willingness to pay for a residential hybrid system that provides heating/cooling and domestic hot water. Renew. Energy.

[33] Lamb N., Elmes D. (2024). Increasing heat pump adoption: analysing multiple perspectives on preparing homes for heat pumps in the UK. Carb. Neutrality.

[34] Brown C., Hampton S., Fawcett T. (2024). Accelerating renewable heat: Overcoming barriers to shared-loop ground source heat pump systems in the United Kingdom. Energy Res. Social Sci..

[35] Davis L.W. (2024). The Economic Determinants of Heat Pump Adoption. Environmental and Energy Policy and the Economy.

[36] Min Y., Mayfield E. (2023). Rooftop solar, electric vehicle, and heat pump adoption in rural areas in the United States. Energy Res. Social Sci..

[37] Shen X., Qiu Y.L., Liu P., Patwardhan A. (2022). The Effect of Rebate and Loan Incentives on Residential Heat Pump Adoption: Evidence from North Carolina. Environ. Resour. Econ..

[38] Mills B., Schleich J. (2012). Residential energy-efficient technology adoption, energy conservation, knowledge, and attitudes: An analysis of European countries. Energy Pol..

[39] Hesselink L.X., Chappin E.J. (2019). Adoption of energy efficient technologies by households–Barriers, policies and agent-based modelling studies. Renew. Sustain. Energy Rev..

[40] Schleich J. (2019). Energy efficient technology adoption in low-income households in the European Union–What is the evidence?. Energy Pol..

[41] Wilson C., Dowlatabadi H. (2007). Models of Decision Making and Residential Energy Use. Annu. Rev. Environ. Resour..

[42] Frederiks E.R., Stenner K., Hobman E.V. (2015). Household energy use: Applying behavioural economics to understand consumer decision-making and behaviour. Renew. Sustain. Energy Rev..

[43] Rao N.D., van Ruijven B.J., Riahi K., Bosetti V. (2017). Improving poverty and inequality modelling in climate research. Nat. Clim. Change.

[44] Wilkerson J.T., Cullenward D., Davidian D., Weyant J.P. (2013). End use technology choice in the National Energy Modeling System (NEMS): An analysis of the residential and commercial building sectors. Energy Econ..

[45] Daioglou V., van Ruijven B.J., van Vuuren D.P. (2012). Model projections for household energy use in developing countries. Energy.

[46] Mastrucci A., van Ruijven B., Byers E., Poblete-Cazenave M., Pachauri S. (2021). Global scenarios of residential heating and cooling energy demand and CO 2 emissions. Climatic Change.

[47] Giraudet L.-G., Guivarch C., Quirion P. (2012). Exploring the potential for energy conservation in French households through hybrid modeling. Energy Econ..

[48] Wilson E.J., Munankarmi P., Less B.D., Reyna J.L., Rothgeb S. (2024). Heat pumps for all? Distributions of the costs and benefits of residential air-source heat pumps in the United States. Joule.

[49] Rai V., Henry A.D. (2016). Agent-based modelling of consumer energy choices. Nat. Clim. Change.

[50] Clarke L., Eom J., Marten E.H., Horowitz R., Kyle P., Link R., Mignone B.K., Mundra A., Zhou Y. (2018). Effects of long-term climate change on global building energy expenditures. Energy Econ..

[51] Cayla J.-M., Maïzi N. (2015). Integrating household behavior and heterogeneity into the TIMES-Households model. Appl. Energy.

[52] Yücel G. (2013). Extent of inertia caused by the existing building stock against an energy transition in the Netherlands. Energy Build..

[53] Snape J.R., Boait P.J., Rylatt R.M. (2015). Will domestic consumers take up the renewable heat incentive? An analysis of the barriers to heat pump adoption using agent-based modelling. Energy Pol..

[54] Niamir L., Filatova T., Voinov A., Bressers H. (2018). Transition to low-carbon economy: Assessing cumulative impacts of individual behavioral changes. Energy Pol..

[55] Niamir L., Kiesewetter G., Wagner F., Schöpp W., Filatova T., Voinov A., Bressers H. (2020). Assessing the macroeconomic impacts of individual behavioral changes on carbon emissions. Climatic Change.

[56] Sopha B.M., Klöckner C.A., Skjevrak G., Hertwich E.G. (2010). Norwegian households’ perception of wood pellet stove compared to air-to-air heat pump and electric heating. Energy Pol..

[57] Sopha B.M., Klöckner C.A., Hertwich E.G. (2013). Adoption and diffusion of heating systems in Norway: Coupling agent-based modeling with empirical research. Environ. Innov. Soc. Transit..

[58] Lee T., Yao R., Coker P. (2014). An analysis of UK policies for domestic energy reduction using an agent based tool. Energy Pol..

[59] Sachs J., Meng Y., Giarola S., Hawkes A. (2019). An agent-based model for energy investment decisions in the residential sector. Energy.

[60] Meles T.H., Ryan L. (2022). Adoption of renewable home heating systems: An agent-based model of heat pumps in Ireland. Renew. Sustain. Energy Rev..

[61] Meles T.H., Ryan L., Mukherjee S.C. (2022). Heterogeneity in preferences for renewable home heating systems among Irish households. Appl. Energy.

[62] Hansen H.H., Korevaar G., Lukszo Z. (2021). The effect of group decisions in heat transitions: An agent-based approach. Energy Pol..

[63] Choi H., Koo Y. (2019). Do I have to buy it now? A vehicle replacement model considering strategic consumer behavior. Transport. Res. Transport Environ..

